# Engineered *Escherichia coli* strains as platforms for biological production of isoprene

**DOI:** 10.1002/2211-5463.12829

**Published:** 2020-03-31

**Authors:** Hyeok‐Won Lee, Jung‐Ho Park, Won‐Kyo Kim, Jin‐Gyeom Lee, Ju‐Seok Lee, Jung‐Oh Ahn, Eun‐Gyo Lee, Hong‐Weon Lee

**Affiliations:** ^1^ Biotechnology Process Engineering Center Korea Research Institute of Bioscience & Biotechnology (KRIBB) Cheongju Korea; ^2^ Bio‐Evaluation Center Korea Research Institute of Bioscience & Biotechnology (KRIBB) Cheongju Korea; ^3^ Department of Bioprocess Engineering University of Science and Technology (UST) of Korea Daejeon Korea

**Keywords:** *Escherichia coli* strains, fermentation, isoprene production, scale‐up

## Abstract

Volatile compounds can be produced by fermentation from genetically engineered microorganisms. *Escherichia coli* strains are mainly used for isoprene production owing to their higher titers; however, this has thus far been confined to only strains BL21, BL21 (DE3), Rosetta, and BW25113. Here, we tested four groups of *E. coli* strains for improved isoprene production, including K‐12 (DH5α, BW25113, W3110, MG1655, XL1‐Blue, and JM109), B [Rosetta (DE3), BL21, and BL21 (DE3)], Crooks C, and Waksman W strains. The isoprene productivity of BL21 and MG1655 was remarkably higher than that of the others in 5‐L fermentation, and scale‐up fermentation (300 L) of BL21 was successfully performed. This system shows potential for biobased production of fuel and volatile compounds in industrial applications.

## Graphical abstract

In this study, we compared 11 engineered recombinant *Escherichia coli* strains for improved isoprene production using one‐ and two‐vector systems. Our results showed that the isoprene productivity of strains BL21 and MG1655 was markedly higher than that of the other strains in 5‐L fermentation. Moreover, we achieved successful scale‐up fermentation using the BL21 strain in a 300‐L fermentor.

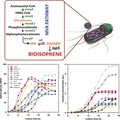

**Abbreviations**
DMAPP: dimethylallyl diphosphate
DO: dissolved oxygen
DXP: 1‐deoxy‐d‐xylulose‐5‐phosphate
*dxr*: DXP reductoisomerase
*dxs*: DXP synthase
IPP: isopentenyl diphosphate
MVA: mevalonate
OD: optical density

Isoprene is an important platform chemical that is widely used in the manufacturing of synthetic rubber along with various other applications such as the production of elastomers and adhesives; moreover, isoprene shows potential to be developed as a fuel additive for gasoline, diesel, or jet fuel [1, 2, 3]. Currently, 800 000 tons of isoprene is produced annually by oil cracking from crude oil refineries. However, the supply of crude oil for isoprene extraction is declining as a result of trends in the petroleum industry toward using lighter hydrocarbon feedstock streams for cracking [4]. As an alternative, biobased isoprene can be successfully synthesized by microbial engineering using the mevalonate (MVA) and 1‐deoxy‐d‐xylulose‐5‐phosphate (DXP) biosynthetic pathways [5, 6, 7].

Several reports have described biobased isoprene production using engineered *Bacillus*, cyanobacteria, and *Saccharomyces cerevisiae* species. For example, the engineered *Bacillus* DSM 10 strain produced 352 μg·L^−1^·optical density (OD)^−1^ of isoprene owing to overexpression of the DXP synthase (*dxs*) and DXP reductoisomerase (*dxr*) genes [8]. Moreover, isoprene titers of 0.32 g·L^−1^ and 37 mg·L^−1^ were also obtained through extensive engineering of *Synechococcus elongatus* and *S. cerevisiae*, respectively [3, 4].

Despite the successful production of isoprene from these engineered microorganisms, the production yield may still be insufficient to meet the future industrial demand. *Escherichia coli* is currently considered the most promising host for producing the highest titers of isoprene, and thus, substantial research attention has focused on the development of various *E. coli* strains for industrial isoprene production. Zhao *et al*. [9] engineered an isoprene synthesis pathway harboring the endogenous *dxs* and *dxr* genes of *E. coli* BL21 (DE3) along with the introduction of the *Populus nigra ispS* gene, which increased isoprene production up to 314 mg·L^−1^. Another study using *E. coli* BL21 (DE3) achieved 6.3 g·L^−1^ isoprene accumulation through heterologous co‐expression of the *Populus* *alba ispS* gene and alteration of the *S. cerevisiae* MVA pathway with a mutation of hydroxymethylglutaryl‐CoA synthase (*mvaS*) [10]. Liu *et al*. [11] produced 20 mg·L^−1^ isoprene from sealed‐bottle fermentation of *E. coli* BL21 (DE3) in which the isopentenyl pyrophosphate isomerase (*idi*) gene was replaced with that from *Streptococcus pneumoniae*. Similarly, Zurbriggen *et al*. [12] reported the production of 320 mg·L^−1^ isoprene from recombinant *E. coli* Rosetta (DE3) harboring the *ispS* gene from *Pueraria montana* along with an exogenous MVA pathway using sealed‐flask cultivation. To date, *E. coli* BL21 is the strain reported to be capable of the highest production of isoprene. Whited *et al*. [13] achieved a high isoprene titer of 60 g·L^−1^ by expression of *P. alba ispS* and the *mvk* gene of the archaea *Methanosarcina mazei* using a combination of the bacterial and yeast MVA pathway from fed‐batch fermentation of *E. coli* BL21. Isoprene has also been produced at a yield of 8.4 g·L^−1^ using *E. coli* BL21 engineered to express a truncated form of *P. alba ispS* along with a gene encoding two types of hydroxy‐2‐methyl‐2‐butenyl‐4‐diphosphate synthase (*ispG*) enzymes in fed‐batch cultivation [14]. We previously obtained 12.7 g·L^−1^ isoprene using *E. coli* DH5α with a two‐vector system of *Populus trichocapa ispS* and the MVA pathway [15]. Despite these numerous reports of enhanced isoprene production using several *E. coli* strains, all of these studies have focused on limited strains, including *E. coli* BL21, BL21 (DE3), BW25113 (DE3), Rosetta (DE3), and DH5α. Although several *E. coli* strains are widely used as hosts for the production of recombinant proteins and metabolites, their performance and stability have not yet been directly compared. Therefore, we considered it necessary to investigate more *E. coli* strains for potential in enhancing isoprene production toward scale‐up industrial application.

Accordingly, in present study, we compared the K‐ and B‐type *E. coli* strains mentioned above, which are typically used for laboratory and industrial purposes, along with the Crooks C and Waksman W strains for their ability of isoprene production, as a representative example of a microorganism‐derived metabolite. The culture conditions for isoprene synthesis were fixed for effective comparison, including agitation, aeration, and consumption of carbon sources using 5‐L batch fermentation. We further examined the ability of the most productive strain for scale‐up isoprene synthesis using a 300‐L fermentor. These findings can help to identify the optimal strain and conditions for improving biobased isoprene synthesis to meet present and future energy demands.

## Materials and methods

### Bacterial strains and plasmids

A total of 11 *E. coli* strains were used for isoprene production: K‐12 (DH5α, BW25113, W3110, MG1655, XL1‐Blue, and JM109), B [Rosetta (DE3), BL21, and BL21 (DE3)], Crooks C, and Waksman W (see Table 1 for strain details). All strains were engineered to express the following six genes of the MVA pathway carried on the pS‐NA plasmid derived from pSTV28, as described previously [16]: *mvaS* and hydroxymethylglutaryl‐CoA reductase (*mvaE*) from *Enterococcus faecalis*; MVA kinase (*mvaK1*), phosphomevalonate kinase (*mvaK2*), and MVA diphosphate decarboxylase (*mvaD*) from *S. pneumoniae*; and *idi* from *E. coli.* Yoon *et al*. [16] suggested that the whole MVA pathway of pS‐NA could provide a sufficient amount of isopentenyl diphosphate (IPP) and dimethylallyl diphosphate (DMAPP). Therefore, the strains were additionally transfected with the plasmid pTS‐sPt‐MVA derived from pTrc99K, encoding isoprene synthase from *Populus trichocarpa* and the MVA pathway operon from pS‐NA [17].

**Table 1:** *Escherichia coli* strains and plasmids used in the study.

*E. coli* strain and plasmid	Genotype or description	Derivation	References
Strains			
DH5α	F^–^ *endA1 glnV44 thi‐1 recA1 relA1 gyrA96 deoR nupG purB20* φ80d*lacZ*ΔM15 Δ(*lacZYA‐argF*)U169, hsdR17(*r_K_* ^–^ *m_K_* ^+^), λ^–^	K‐12	
BW25113	F^−^, DE(araD‐araB)567, lacZ4787(del)::rrnB‐3, LAM^−^, rph‐1, DE(rhaD‐rhaB)568, hsdR514	K‐12	
W3110	F^−^ λ^−^ rph‐1 INV(rrnD, rrnE)	K‐12	
MG1655	K‐12 F^–^ λ^–^ *ilvG* ^–^ *rfb‐50 rph‐1*	K‐12	
XL1‐Blue	endA1 gyrA96(nal^R^) thi‐1 recA1 relA1 lac glnV44 F'[ ::Tn10 proAB^+^ lacI^q^ Δ(lacZ)M15] hsdR17(r_K_^−^ m_K_^+^)	K‐12	
JM109	endA1 glnV44 thi‐1 relA1 gyrA96 recA1 mcrB^+^ Δ(lac‐proAB) e14‐ [F' traD36 proAB^+^ lacI^q^ lacZΔM15] hsdR17(r_K_^−^m_K_^+^)	K‐12	
Rosetta (DE3)	F^–^ *ompT gal dcm‐1 hsdS_B_*(*r_B_* ^–^ *m_B_* ^–^) λ(DE3 [*lacI lacUV5*‐*T7p07 ind1 sam7 nin5*]) [*malB* ^+^]_K‐12_(λ^S^) pLysSRARE[*T7p20 ileX argU thrU tyrU glyT thrT argW metT leuW proL ori*_p15A_](Cm^R^)	B	
BL21	F‐ dcm ompT hsdS(rB‐ mB‐) gal [malB+]K‐12(λS)	B	
BL21 (DE3)	F^–^ *ompT gal dcm lon hsdS_B_*(*r_B_* ^–^ *m_B_* ^–^) λ(DE3 [*lacI lacUV5*‐*T7p07 ind1 sam7 nin5*]) [*malB* ^+^]_K‐12_(λ^S^)	B	
Crooks strain C	Wild‐type	C	
Waksman strain W	Wild‐type	W	
Plasmids			
pTS‐sPt‐MVA	pTrc99K containing *mvaE* and *mvaS* from *Enterococcus faecalis*; *mvaK1, mvaK2*, and *mvaD* from *S. pneumoniae*; *idi* from *E. coli*; and *ispS* from *Populus trichocarpa* 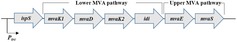	pTrc99A	[16]
pS‐NA	pSTV28 containing *mvaE* and *mvaS* from *Enterococcus faecalis*; *mvaK1, mvaK2,* and *mvaD* from *S. pneumoniae*; and *idi* from *E. coli* 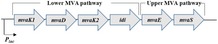	pSTV28	[17]

### Culture conditions

Transformed *E. coli* strains for isoprene production were plated on LB agar plates supplemented with 50 µg·mL^−1^ each of chloramphenicol and kanamycin as required. A single colony grown on the agar plate was transferred to a 1‐L Erlenmeyer flask containing 200 mL of LB broth as the seed culture for 5‐L fermentation. For scale‐up 300‐L fermentation, the seed flask was transferred to a 30‐L fermentor containing 10 L of LB broth as a seed culture. After 6 h of cultivation at 37 °C on a rotary shaker (200 r.p.m.), the culture was used as the seed culture, which was inoculated into a 5‐L or 300‐L fermentor (KFC LA‐150; Kobiotech Co., Ltd., Incheon, Korea) containing 100 L of the initial medium (10 g·L^−1^ glycerol, 20 g·L^−1^ yeast extract, 10 g·L^−1^ casein peptone, 5 g·L^−1^ (NH_4_)_2_SO_4_, 3 g·L^−1^ KH_2_PO_4_, 3 g·L^−1^ Na_2_HPO_4_, 1 g·L^−1^ MgSO_4_·7H_2_O, 0.4 mL·L^−1^ antifoam, 50 μg·mL^−1^ kanamycin, and 50 μg·mL^−1^ chloramphenicol) and 1 mL of a trace element solution in 1 N HCl (13.2 g·L^−1^ CaCl_2_·2H_2_O, 8.4 g·L^−1^ FeSO_4_·7H_2_O, 2.4 g·L^−1^ MnSO_4_·4H_2_O, 2.4 g·L^−1^ ZnSO_4_·7H_2_O, 0.48 g·L^−1^ CuSO_4_·5H_2_O, 0.48 g·L^−1^ CoCl_2_·6H_2_O, 0.24 g·L^−1^ Na_2_MoO_4_·2H_2_O, and 0.06 g·L^−1^ K_2_B_4_O_7_·XH_2_O) for batch or fed‐batch cultivation. The phosphate‐containing compounds (KH_2_PO_4_ and Na_2_HPO4) were sterilized separately from the main medium. To identify the optimal culture conditions, aeration was maintained at 1 vvm until the end of culturing and the initial glycerol concentration was maintained as high as 50 g·L^−1^. The stirring speed was gradually increased to 300–1100 r.p.m. to maintain the dissolved oxygen (DO) concentration at ≥ 20% by adjusting the rate of agitation and maintaining the pH at 7.0 with 10 N NaOH. Incubation continued until the glycerol was exhausted and isoprene production no longer increased. For 5‐L fed‐batch cultivations using the pTS‐sPt‐MVA system, the seed culture (OD_600_ = 4.3) was first incubated for 12 h, and then, culturing was carried out for 54 h using a feeding medium comprising 80 g·L^−1^ of yeast extract and 800 g·L^−1^ of glycerol. The initial feed rate was 6 g·L^−1^·h^−1^, which was optimal at 9.5 h following the initial incubation. The feeding rate was continuously adjusted using a stepwise strategy to ensure that the glycerol was not depleted. Aeration was increased to 2 vvm at 22 h of culture, and agitation was also gradually increased to minimize the depletion of DO. For 300‐L fed‐batch cultivation, liquid oxygen was initiated at 22 h of cultivation to prevent the depletion of DO during culturing. The oxygen supply was introduced at < 10% of aeration, but was gradually increased to maintain the concentration of DO between 20% and 40%, since the increase in DO concentration by changing the aeration rate and stirrer speed has been shown to improve the yield of biomass on the substrate. To overcome the loss of performance of a scale‐up fermentor such as impeller tip speed and mixing time, many researchers have used pure oxygen to maintain the DO level in the culture broth [18, 19]. The continuous feed medium was composed of 800 g·L^−1^ glycerol and 80 g·L^−1^ yeast extract, and was fed into the fermentor by a peristaltic pump to obtain an appropriate feeding rate.

### Analytical methods

An autosampler (Locas, Daejeon, Korea) was used to measure both the OD and the glycerol concentration in the culture broth. Cell growth was monitored by measuring the OD at 600 nm (OD_600_) using a spectrophotometer (Uvikon 941 Plus; Kontron Instruments Co., Zurich, Switzerland). The cell dry weight was determined using a predetermined conversion factor of 0.3 g cell dry weight·L^−1^·OD^−1^ [20]. The residual glycerol concentration was analyzed on a HPLC system with an RID detector (RID‐7515A; ERC Instrument Co., Kawaguchi, Japan) equipped with an Aminex 87H ion‐exclusion column (Bio‐Rad, Hercules, CA, USA). The column temperature was maintained at 85 °C, and the mobile phase was deionized water applied at a flow rate of 0.5 mL·min^−1^. The levels of the by‐products acetate and lactate in the culture broth were also analyzed with HPLC at a detection wavelength of 210 nm with an ion‐exchange HPLC column (Aminex HPX‐87H, 7.8 × 300 mm; Bio‐Rad). The isoprene concentration was measured with a gas chromatograph (Varian X‐3300, Agilent Technolgies, Santa Clara, CA, USA) equipped with a flame ionization detector. To increase the detection level of isoprene, a 1‐mL sample was injected into an Agilent J&W GC column (30 m × 0.53 mm internal diameter). We adopted the novel online monitoring system developed in our previous study using gas chromatography for the analysis of isoprene production during aerobic fermentation [15]. The temperature program used was 3 min at 50 °C followed by an increase to 150 °C for 10 min; the column was maintained at this temperature for 12 min before lowering to 50 °C again.

## Results

### Comparison of the isoprene production of *E. coli* strains in 5‐L batch cultivation

**Fig. 1:**
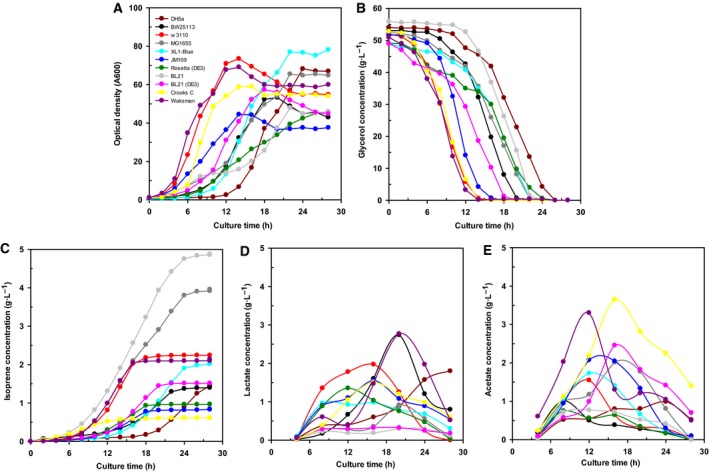
Time course of (A) cell mass, (B) glycerol consumption, (C) isoprene concentration, (D) lactate concentration, and (E) acetate concentration during 5‐L batch fermentation of recombinant *Escherichia coli* strains harboring pTS‐sPT‐MVA.

As shown in Fig. 1A, maximal cell growth of the wild‐type strain W3110 (OD_600_ = 73.5) was observed at 14 h of incubation, whereas the JM109 strain exhibited the lowest growth rate of all strains, with an OD_600_ value of 46 at 28 h of culture.

The cultivation results of all *E. coli* strains are shown in Table 2. The maximum growth rate (μ_max_·h^−1^) was the highest in strain W3110 and was the lowest in the Rosetta (DE3) strain. The lag phase lasted from 6 to 10 h for XL1‐Blue and DH5α, respectively. As shown in Fig. 1B, strains W3110 and DH5α consumed the glycerol in association with growth after 14 and 26 h of cultivation, respectively.

**Table 2:** Results of batch cultivation by four types of *Escherichia coli* strains.

	Strains	Culture time (h)	Consumed glycerol (g·L^−1^)	Specific growth rate (μ_max_)	*X*_max_ ^a^ (g·L^−1^)	*P*_max_ ^a^ (g·L^−1^)	*Q*_p_ (mg·L^−1^·h^−1^)	*Y*_p/x, max_ (mg·g cells^−1^)	*Y*_p/s_ (mg·g carbon sources^−1^)
K‐12	DH5α	28	54	0.59	20.40 ± 0.12	1.45 ± 0.18	51.67	70.92	26.79
	BW25113	26	53	0.35	15.99 ± 0.08	1.41 ± 0.12	54.10	87.97	26.54
	W3110	23	52	0.79	22.05 ± 0.30	2.24 ± 0.07	97.39	101.59	43.08
	MG1655	25	52.6	0.40	19.65 ± 0.06	3.90 ± 0.32	156.00	198.47	74.14
	XL1‐Blue	25	49.1	0.44	14.37 ± 0.16	2.02 ± 0.25	80.80	140.57	41.14
	JM109	21	51.6	0.72	13.35 ± 0.32	0.82 ± 0.19	39.05	61.42	15.89
B	Rosetta (DE3)	28	49.05	0.30	13.80 ± 0.28	0.96 ± 0.32	34.29	69.57	19.57
	BL21	25	55.8	0.46	14.37 ± 0.22	4.84 ± 0.20	193.60	336.81	86.74
	BL21 (DE3)	21	49	0.37	17.22 ± 0.16	1.51 ± 0.07	72.06	87.88	30.88
C	*Crooks* C	22	52.8	0.76	17.64 ± 0.03	0.61 ± 0.14	27.88	34.77	11.62
W	*Waksman* W	20	50.92	0.77	20.73 ± 0.15	2.09 ± 0.06	104.67	100.98	41.11

^a^ Data are mean ± SD; *n* = 3 per strain.

*Escherichia coli* MG1655 and BL21 demonstrated the highest isoprene productivity at 26 h of culture, respectively, whereas the Crooks C strain exhibited the lowest value. Productivity per hour (*Q*_p_) was also the highest in BL21 and lowest in Crooks C. Isoprene production yield per unit cell was also substantially higher in BL21 than that in Crooks C. Strain BL21 also showed the highest yield of produced isoprene vs. consumed glycerol, while MG1655 had the lowest level (Fig. 1C, Table 2).

Organic acids, which gradually increase from the beginning of culture, tend to be consumed again once the carbon source is depleted. The production of lactate and acetate was < 1 g·L^−1^ lower than in the BL21 strains, while that of Waksman W and DH5α reached about 3.0 g·L^−1^ in the late stage of culture (Fig. 1D,E).

### *Escherichia coli* BL21 and MG1655 in 5‐L fed‐batch cultivation

Since *E. coli* BL21 and MG1655 demonstrated the highest isoprene production levels using the single‐vector pTS‐sPt‐MVA under the batch culture system, these strains were selected as candidate production strains for investigating the reproducibility of the isoprene production process by fed‐batch culture.

**Fig. 2:**
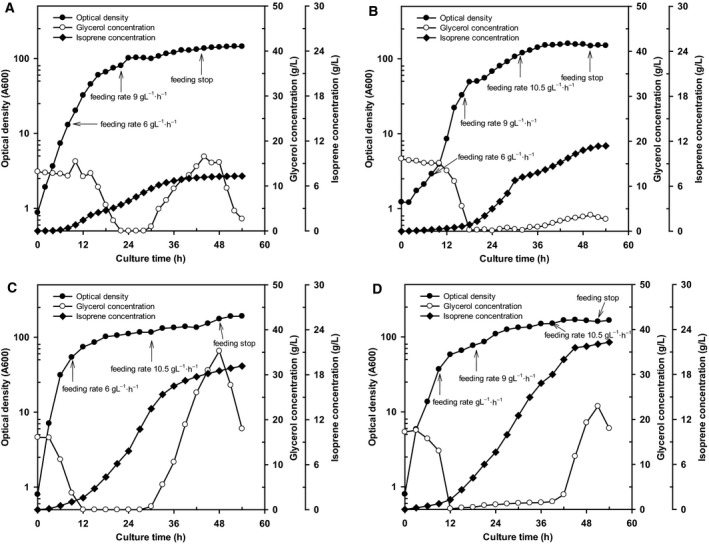
Time course of (A) *Escherichia coli* MG1655 harboring pTS‐sPT‐MVA, (B) *E. coli* MG1655 harboring pTS‐sPT‐MVA and pS‐NA, (C) *E. coli* BL21 harboring pTS‐sPT‐MVA, (D) and *E. coli* BL21 harboring pTS‐sPT‐MVA and pS‐NA during 5‐L fed‐batch fermentation.

The yields of cell mass, specific growth rate, and isoprene production of *E. coli* strains MG1655 and BL21 are summarized in Table 3. As shown in Fig. 2A,B, maximal cell growth of *E. coli* MG1655 and BL21 reached an OD_600_ of 164 at 52 h of culture and of 159.4 at 44 h of culture, respectively, and the specific growth rates were similar. For MG1655, cell growth continued after 30 h of incubation; however, isoprene productivity exhibited a rapid decrease despite the addition of feeding medium. Overfeeding of the feeding medium led to an accumulation of 16 g·L^−1^ of the carbon source in the culture broth at 42 h. Overall, strain BL21 showed 4.06 g·L^−1^ higher isoprene production than MG1655 in fed‐batch culture.

**Table 3:** Results of fed‐batch cultivation by *Escherichia coli* MG1655 and BL21.

Strains	Culture type	Culture time (h)	Consumed glycerol (g·L^−1^)	Specific growth rate (μ_max_)	*X*_max_ ^a^ (g·L^−1^)	*P*_max_ ^a^ (g·L^−1^)	*Q*_p_ (mg·L^−1^·h^−1^)	*Y*_p/x, max_ (mg·g cells^−1^)	*Y*_p/s_ (mg·g carbon sources^−1^)
MG1655 harboring pTS‐sPT‐MVA	5 L	54	181	0.45	43.5 ± 0.28	7.32 ± 0.09	135.55	168.27	40.44
BL21 harboring pTS‐sPT‐MVA	5 L	54	161	0.48	45.3 ± 0.32	11.38 ± 0.16	210.74	251.21	70.68
MG1655 harboring pTS‐sPT‐MVA and pS‐NA	5 L	54	223	0.49	56.7 ± 0.13	19.16 ± 0.38	354.81	337.91	85.91
BL21 harboring pTS‐sPT‐MVA and pS‐NA	5 L	54	198	0.44	49.8 ± 0.08	22.29 ± 0.24	412.77	447.59	112.57
BL21 harboring pTS‐sPT‐MVA and pS‐NA	300 L	72	241	0.53	33.6 ± 0.17	25.2 ± 0.15	350	750	104.56

^a^ Data are mean ± SD; *n* = 3 per strain.

Figure 2C and Table 3 show the results of the 5‐L fed‐batch culture of *E. coli* MG1655 using a two‐vector system (pTS‐sPt‐MVA and pS‐NA). Maximal cell growth reached an OD_600_ of 190 at 54 h of culture. The maximum yield of isoprene was 11.38 ± 0.16 g·L^−1^ higher than that obtained using the single‐vector system. In addition, the *Q*_p_ of isoprene, the production yield of isoprene per unit cell, and the production yield of isoprene relative to the consumed glycerol were all higher than those obtained with the single‐vector system. Similarly, *E. coli* BL21 harboring pTS‐sPt‐MVA and pS‐NA reached maximal growth with an OD_600_ of 168.8 at 45 h of culture (Fig. 2D). However, the feeding medium comprising glycerol and yeast extract at 6 g·L^−1^·h^−1^ failed to ensure that the carbon source concentration remained at ≥ 20 g·L^−1^ in the culture medium following 42 h of cultivation, and although more glycerol was consumed than under the single‐vector condition, this amount was lower than that consumed by strain MG1655. However, the isoprene production was higher at 54 h of culture despite an excessive supply of feeding media. The *Q*_p_ of isoprene, the production yield of isoprene per unit cell, and the production yield of isoprene relative to the consumed glycerol were 714 mg·L^−1^·h^−1^, 441 mg·g cells^−1^, and 195 mg·g^−1^ carbon source, respectively.

### *Escherichia coli* BL21 in 300‐L fed‐batch cultivation for isoprene production using the two‐vector system

**Fig. 3:**
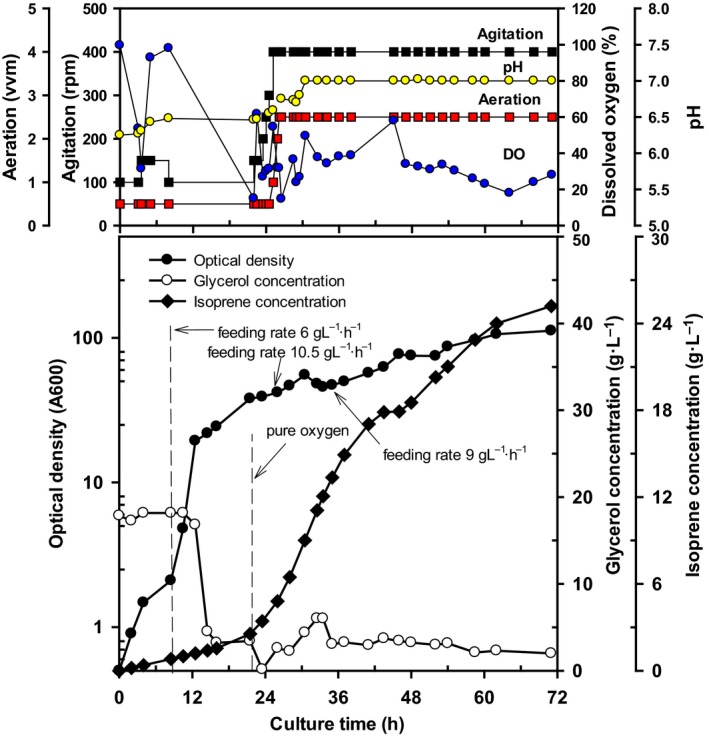
Time course of 300‐L fed‐batch cultivation of *Escherichia coli* BL21 harboring pTS‐sPT‐MVA and pS‐NA.

Based on the results of 5‐L fermentation, we tested a 300‐L fermentor as a pilot study for scaling up isoprene production using *E. coli* BL21 containing both plasmids pTS‐sPt‐MVA and pS‐NA. As shown in Fig. 3, maximal cell growth and the specific growth rate (μ_max_·h^−1^) of OD_600_ = 112 and 0.53, respectively, were observed at 72 h. The total consumed glycerol was higher than that observed under any other condition for either strain. The feeding rate was gradually increased using a stepwise gradient according to cell growth. The DO appeared to become depleted from 11.5 to 28 h of cultivation, and isoprene productivity was also low during this period (data not shown). In the 300‐L scale‐up fermentation, the isoprene production and *Q*_p_ were at the highest levels observed in any other condition. In addition, the production yield of isoprene per unit cell and production yield of isoprene relative to the consumed waste glycerol were also the highest observed (Table 3).

## Discussion

*Escherichia coli* is a popular host for biotechnological applications; however, only four common laboratory strains (K, B, C, W, and their derivatives) are listed in biological safety guidelines [21, 22]. In this study, we tested 11 *E. coli* strains as representatives of the wild‐types and their derivatives for comparison of volatile isoprene production. In particular, strains K and B are the most widely used *E. coli* strains for overproducing recombinant proteins and various bioproducts at the industrial scale [23, 24]. Since *E. coli* Crooks C was first sequenced in 2007, it has also been used to produce a variety of bioproducts [25]. In addition, *E. coli* Waksman W entered the spotlight as the standard strain for sensitivity assays to streptomycin and other antibiotics [26]. Thus, we first carried out a simple 5‐L batch cultivation without specific feeding strategies to compare isoprene synthesis by recombinant *E. coli* strains. This represents the first assessment of isoprene production in strains other than the primary four common laboratory types.

Consistently, in the present study, we showed that the *E. coli* wild‐type strains W3110, Crooks C, and Waksman W achieved relatively higher growth rates and organic acid accumulation, but tended to have lower isoprene production than the other strains tested. Isoprene is synthesized from acetyl‐CoA by eight enzymatic steps using the MVA pathway. Acetoacetyl‐CoA is formed from two acetyl‐CoA moieties by a biosynthetic β‐ketothiolase [5]. Thus, the lower isoprene production in these strains could be attributed to preferential flow of acetyl‐CoA for the tricarboxylic acid cycle [27]. By contrast, wild‐type *E. coli* BL21 (4.84 ± 0.20 g·L^−1^) and MG1655 (3.90 ± 0.32 g·L^−1^) showed dramatically greater isoprene productivity than the other strains in 5‐L batch cultivation, which could be attributed to the allocation of excessive acetyl‐CoA to isoprene, which would otherwise form growth‐inhibitory organic acids such as lactate and acetate. Indeed, organic acid accumulation is one of the major problems encountered during cultivation of *E. coli* because it inhibits cell growth and production of foreign proteins [28]. Therefore, the low isoprene productivity of strains Crooks C (0.61 g·L^−1^) and Waksman W (2.09 g·L^−1^) is attributed to their higher levels of lactate and acetate production (Fig. 1D,E).

The production of isoprene was further improved in *E. coli* MG1655 and BL21 using 5‐L fed‐batch cultivation and a two‐vector system (pTS‐sPt‐MVA and pS‐NA). This improvement is considered to be derived from the increased supply of IPP and DMAPP from pS‐NA through augmentation of the MVA pathway, resulting in high levels of isoprenoid compounds such as isoprene and lycopene [16, 17]. Based on these results, we further tested a 300‐L scale‐up fermentation process with the two‐vector system using *E. coli* BL21 for 72‐h cultivation to improve isoprene production. This resulted in the highest level of isoprene synthesis observed under all conditions tested, with 25.2 ± 0.15 g·L^−1^ isoprene produced by the end of culture.

In summary, we have demonstrated that *E. coli* MG1655 and BL21 are suitable strain choices for the production of isoprene during batch fermentation. In particular, 300‐L scale‐up fermentation was successfully achieved using pure oxygen to protect against the depletion of DO with *E. coli* BL21 harboring a two‐vector system. This system is particularly advantageous for practical applications in that it is easily adapted for the detection of various volatile organic compounds and volatile gas production using microbial‐based fermentation.

## Conflict of interest

The authors declare no conflict of interest.

## Author contribution

Hy‐WL, J‐HP, and Ho‐WL conceived and designed the experiments. Hy‐WL, W‐KK, and J‐GL performed the experiments. J‐SL, J‐OA, and E‐GL performed the data analysis. Hy‐WL, J‐HP, and Ho‐WL analyzed the data and wrote the paper.
